# A Review of Endoscopic Ultrasound-Guided Chemoablative Techniques for Pancreatic Cystic Lesions

**DOI:** 10.3390/diagnostics13030344

**Published:** 2023-01-17

**Authors:** Bryn Koehler, Da Yeon Ryoo, Somashekar G. Krishna

**Affiliations:** 1 Department of Internal Medicine, Ohio State University Wexner Medical Center, Columbus, OH 43210, USA; 2 Division of Gastroenterology, Department of Internal Medicine, Ohio State University Wexner Medical Center, Columbus, OH 43210, USA

**Keywords:** pancreatic cystic lesions, intraductal papillary mucinous neoplasms, mucinous cystic neoplasms, pancreatic cancer, EUS-FNI, EUS-guided chemoablation, LSAM-PTX, EUS-nCLE

## Abstract

Pancreatic cystic lesions (PCLs) are known precursors to pancreatic cancer, one of the deadliest types of cancer worldwide. Surgical removal or pancreatectomies remain the central approach to managing precancerous high-risk PCLs. Endoscopic ultrasound (EUS)-guided therapeutic management of PCLs is a novel management strategy for patients with prohibitive surgical risks. Various ablation techniques have been explored in previous studies utilizing EUS-guided fine needle injection (FNI) of alcohol and chemotherapeutic agents. This review article focuses on EUS-FNI and chemoablation, encompassing the evolution of chemoablation, pancreatic cyst selection, chemotherapy drug selection, including novel agents, and a discussion of its safety and efficacy.

## 1. Introduction

Pancreatic malignancy is one of the deadliest types of cancer worldwide. With an 11.5% 5-year survival, it is responsible for approximately 8.2% of all cancer-related deaths in the US [1]. Globally, it is the seventh-leading cause of cancer-related death, and by 2030, it will be the second-leading cause of cancer-related death in the US [2]. While numerous established risk factors predict the development of pancreatic cancer, including older age, male sex, diabetes mellitus, obesity, and diets high in fats, the two most dominant are cigarette smoking and family history. The lack of an adequately specific screening test for the general population contributes to delayed diagnoses and increased mortality. Pancreatic cystic lesions (PCLs) are known precursors to pancreatic cancer and provide an opportunity for early intervention to prevent progression to pancreatic malignancy.

The prevalence of PCLs has been increasing rapidly in recent years, largely due to improvements in cross-sectional imaging and an aging population [3]. These heterogeneous lesions are often discovered incidentally and pose significant uncertainty in management due to their varying malignant potential, with over 50% of incidentally discovered PCLs being premalignant or malignant [4]. Accurate diagnosis of PCL type and risk stratification of premalignant lesions is crucial in determining whether observation or surgical resection is most appropriate. In patients with prohibitive surgical risks, local ablation of the PCL can be attempted.

Neoplastic PCLs have two main subtypes—mucinous and non-mucinous. Mucinous PCLs include intraductal papillary mucinous neoplasms (IPMNs) and mucinous cystic neoplasms (MCNs). Non-mucinous PCLs include serous cystic neoplasms (SCNs), solid pseudopapillary neoplasms (SPNs), and cystic neuroendocrine neoplasms (cystic-NETs). SCNs are the most common benign pancreatic neoplasm, with a malignant transformation rate of 1 to 3% [5,6]. SPNs are most commonly seen in young females and make up 0.3 to 2.7% of all pancreatic tumors. Margin-negative surgical resection is generally curative; however, these lesions do have the potential to recur or metastasize [7]. Pancreatic NETs arise from the islets of Langerhans and have two main subtypes—cystic and solid. Cystic-NETs make up 6.5–36.1% of all pancreatic NETs and are considered to have less aggressive behavior than solid-NETs [8]. Pseudocysts are the most common non-neoplastic PCLs [9]. They are inflammatory lesions that result from leakage of pancreatic fluid in the setting of acute or chronic pancreatitis [10]. Unlike true cysts or cystic neoplasms, they lack an epithelial lining and may spontaneously resolve.

Among all PCLs, IPMNs and MCNs carry the greatest malignant potential. IPMNs are characterized by the proliferation of mucin-producing columnar cells in the main pancreatic duct or side branches, leading to dilatation of the duct and its proximal segment. While estimates of the incidence of IPMNs vary, a case series of 401 patients with surgically-resected PCLs demonstrated that IPMN was the most common diagnosis, with 25% of PCLs being main-duct IPMN (MD-IPMN) and 23% being branch-duct IPMN (BD-IPMN) [11]. The mean frequency of malignancy is 61.6% in MD-IPMN and 25.5% in BD-IPMN [12]. MCNs share a composition of mucin-producing epithelium but, unlike IPMNs, do not communicate with the pancreatic duct. These account for about 10% of all PCLs and are predominantly seen in women. The same surgical case series cited above estimates that MCNs make up approximately 11 to 18% of surgically-resected PCLs [11]. The risk of high-grade dysplasia or adenocarcinoma in MCN is approximately 10–39% [13]. Historically, these mucinous PCLs have been managed with surgical resection; however, these attempts at curative treatment pose significant risks. Pancreatectomy has a morbidity rate of up to 40% and a mortality rate ranging from 1–2% [14,15,16]. In patients deemed high risk for surgery, newer EUS-guided ablative techniques are being explored. 

Endoscopic ultrasound (EUS) has evolved in recent years from a primary diagnostic tool to a therapeutic one. EUS fine needle injection (EUS-FNI) has several uses in pancreatic cancer patients, including EUS-guided cholangiopancreatography for biliary drainage and stent placement, facilitating difficult endoscopic retrograde cholangiopancreatography (ERCP) via injection of contrast or dyes, marking, or tattooing pancreatic lesions and lymph nodes for surgical or radiation planning, and managing cancer-related pain by neurolysis with direct injection of bupivacaine and ethanol [17]. EUS-FNI is now being harnessed as a method of delivering antitumor agents directly into PCLs. 

## 2. EUS-Guided Ethanol Ablation

The discovery of EUS-FNI opened the door to the potential to utilize the technique in managing PCLs. The first of those to explore EUS-FNI in treating PCLs was Gan et al., who aspirated the lesion with a needle and lavaged the cyst cavity with ethanol [18]. A total of 25 patients were treated with ethanol lavage, 23 patients completed follow-up at 6–12 months, and 8 patients (35%) experienced complete cyst ablation. Although this study in and of itself did not show a remarkable resolution rate in PCL with ethanol lavage, it was a milestone study for many reasons. Firstly, it demonstrated that ethanol lavage of PCLs with EUS-FNI can be safely conducted without significant adverse events. Secondly, it proved the feasibility of EUS-FNI to be used in treating PCLs. Following this monumental study, multiple investigators lavaged PCLs using varying concentrations of EtOH (80–99%) and saw up to 85% of the complete resolution of PCLs [19,20,21,22]. However, these follow-up studies also revealed some clinically significant adverse events associated with ethanol lavage. Dewitt et al. reported that 20% of patients experienced abdominal pain after the procedure, and 4% acquired post-ablation pancreatitis [19]. The etiology of acute pancreatitis was thought to be secondary to the extravasation of ethanol out of the cyst into the pancreatic parenchyma and duct, causing inflammation.

## 3. EUS-Guided Chemoablation and Inclusion Criteria 

Table 1 lists several relevant studies in EUS-guided chemoablation of PCLs, highlighting enrollment data, response rate, adverse events, and study conclusions. The first group to explore alternative injectables to achieve an increased rate of complete resolution of PCLs with EUS-FNI was Oh et al. [23]. Their choice of agent was paclitaxel, a chemotherapeutic agent that inhibits cell replication. Based on how local injection chemotherapy is used to treat localized tumors in other organ systems, such as endobronchial lesions of lung cancer and advanced ovarian cancer, Oh et al. hypothesized that paclitaxel injection into the cyst cavity would eradicate the cyst. Oh et al. employed the following inclusion criteria: (1) unilocular or oligolocular cystic tumors (oligolocular cyst was defined as having two to six locules within a cyst in a later study by Oh et al.), (2) indeterminate cystic neoplasms despite evaluation by EUS-FNA, and (3) cystic tumors that increased in size during the observation period [23,24]. Moyer et al. used similar inclusion criteria, although they defined the eligible PCL size to be between 1 and 5 cm [25]. Oh et al. defined their exclusion criteria as (1) cystic tumors that had the typical morphology of serous cystadenomas and pseudocysts (therefore including only mucinous and indeterminate cysts), (2) evidence of communication between the PCL and the main pancreatic duct, (3) overt carcinomas with peripancreatic invasion, and (4) patients with a bleeding tendency (pro-thrombin time >1.5 international normalized ratio or a platelet count <50,000/microliter) [23]. Interestingly, DeWitt et al. did not consider communication between the PCL and the main pancreatic duct as an exclusion criterion, while Moyer et al. did. While both Oh et al. and Moyer et al. excluded serous cystadenoma and pseudocysts, DeWitt et al. enrolled all benign PCLs. Choi et al., who implemented similar inclusion and exclusion criteria as those of Moyer et al., excluded only pseudocysts, including mucinous, serous, and indeterminate PCLs [26]. DeWitt et al. and Moyer et al. further honed the exclusion criteria to include pregnancy, incarceration, inability to provide informed consent, unacceptably high risk for deep sedation, active acute pancreatitis (lipase greater than 3 times the upper limit of normal), CA 19–9 greater than 40 U/mL, pancreatic necrosis, antiplatelet medications and anticoagulants, hematocrit less than 30, dilated main pancreatic duct by previous imaging, ascites, portal hypertension, suspicious liver or pulmonary lesions, or distant enlarged lymph nodes [25,27]. 

## 4. Cyst Selection 

Given the variation in pancreatic cysts discussed in the introduction, the question of which types of PCLs would respond best to chemoablation has become central to the investigation of this technique. As discussed previously, each group of researchers utilized varying inclusion criteria for the PCLs they would treat with EUS-FNI chemoablation. The general consensus was the first to treat cystic lesions that are not currently of malignant quality, since those cysts would be better candidates for surgical removal. Additionally, if the patients were not good surgical candidates, EUS-FNI chemoablation could be considered. The primary purpose of EUS-FNI chemoablation is to prevent PCLs from evolving into overt malignancy. Therefore, studies have found it most effective to ablate cysts that can potentially turn malignant during the disease process, specifically IPMNs and MCNs. Indeterminate PCLs have also been included in the studies due to their unknown potential for malignancy.

Historically, the method of classifying PCLs to evaluate candidacy for EUS-FNI chemoablation has been with cross-sectional imaging and cystic fluid studies. PCLs with a honeycomb appearance on imaging were identified as SCAs, and those with parenchymal changes on imaging could be identified as pseudocysts. Cyst sizes were calculated based on cross-sectional imaging as well, both to evaluate inclusion and to determine the response to therapy. Cyst fluid was aspirated for analysis prior to ablation. Moyer et al. classified MCN as lesions with CEA greater than 200 ng/mL and amylase less than 800 U/L and IPMN as lesions with CEA greater than 200 ng/mL and amylase greater than 800 U/L [25]. Oh et al. classified SCA as lesions with CEA less than 5 ng/mL and pseudocysts when CEA was less than 5 ng/ML but amylase greater than 800 U/L. Lesions with fluid studies that did not meet any of these criteria were classified as indeterminate [23]. FNA of the cystic component and evaluation of its cytology lend a sensitivity of 51% and specificity of 94% for the diagnosis of malignant pancreatic cystic lesions, with its lower sensitivity due to sampling error in meta-analyses [31,32].

EUS-guided needle-based confocal laser endomicroscopy (EUS-nCLE) allows real-time microscopic visualization of the PCL epithelium [33]. In addition to providing an accurate diagnosis of PCLs, EUS-nCLE has been studied for differentiating IPMNs with high-grade versus low-grade dysplasia [33,34]. Cyst fluid molecular analysis by next-generation sequencing analysis allows accurate diagnosis of cyst type and also reliably identifies IPMNs with advanced neoplasia [35,36]. Most of the published studies on EUS-guided chemoablation have yet to utilize accurate diagnostic tools, such as EUS-nCLE and cyst fluid molecular analysis, in PCL diagnosis and risk stratification.

Additional diagnostic measures with EUS include contrast-enhanced EUS (CE-EUS) and through-the-needle biopsy (TTNB). CE-EUS can distinguish mural nodules from mural clots in IPMNs and can potentially identify high-grade dysplasia or invasive carcinoma among mural nodules [37,38]. Lastly, TTNB can offer higher quality histological samples than FNA to offer a more accurate diagnosis of PCLs [39].

## 5. Chemotherapy Agent Selection, Efficacy, and Safety

There have been a number of anti-tumor agents, including alcohol, oncolytic viruses, brachytherapy, and chemotherapy drugs, trialed for targeted treatment of PCLs using the EUS-FNI technique [40]. The primary chemotherapeutic agents used for the management of PCLs are paclitaxel and gemcitabine, sometimes in combination with each other or preceded by alcohol lavage. 

Paclitaxel binds to tubulin and promotes the formation of aberrant mitotic spindles that disrupt mitosis. Its hydrophobic nature makes it an ideal candidate for intralesional delivery without significant extravasation [41]. Various formulations of injectable paclitaxel for intralesional delivery have been tested in animal models and have demonstrated high, sustained local concentrations of the drug without significant extravasation into the surrounding tissue [42]. Therefore, it has been the chemotherapeutic agent of choice in the vast majority of studies evaluating the chemoablation of PCLs. A second agent, gemcitabine, has also been used in combination with paclitaxel in some studies. Gemcitabine is a nucleoside analog that promotes apoptosis of rapidly dividing malignant cells [43]. The two agents synergize in animal models, as paclitaxel reduces cytidine deaminase, the primary enzyme that metabolizes gemcitabine [44]. 

Five studies have assessed the safety and efficacy of ethanol lavage with paclitaxel injection in the management of PCL. Of the 256 PCLs treated in these studies, 170 (66%) were completely resolved, and 46 (18%) were partially resolved on follow-up imaging [23,24,26,27,45]. DeWitt et al. also reported the presence of K-ras mutations in aspirated cyst fluid, and noted elimination of these mutations in 8 of 11 PCLs (73%) with baseline mutations after EUS-guided ethanol lavage with paclitaxel injection (EUS-EP) [27]. The most commonly reported procedure-related adverse events were abdominal pain (*n* = 4, 2%), pancreatitis (*n* = 12, 5%), hyperamylasemia (*n* = 6, 2%), and fever without bacteremia (*n* = 2, <1%) [23,24,26,27,46]. The only predictors of cyst resolution were a cyst diameter <35 mm and volume <22 mL [45]. A systematic review conducted to evaluate the safety and efficacy of alcohol lavage or paclitaxel-based pancreatic cyst ablation saw complete cyst resolution in 32.8% of patients treated with alcohol lavage and in 63.6% of patients treated with paclitaxel-based cyst ablation [46]. Post-ablation adverse events were documented for alcohol lavage and paclitaxel-based cyst ablation were 21.7% and 15%, respectively. Choi et al. established the durability of this technique, as only 2 lesions out of 114 cysts that demonstrated complete resolution had any evidence of recurrence at 72 months [26].

Subsequently, Moyer et al. questioned the necessity of ethanol in chemoablation with paclitaxel. The initial study enrolled 10 patients, with 4 receiving ethanol lavage with paclitaxel injection and 6 receiving normal saline with paclitaxel injection. It established the non-inferiority of ethanol-free chemoablation with paclitaxel, as the ethanol-free arm had a complete resolution in 67% of patients, and the ethanol arm had a complete resolution in 75%. The only procedure-related adverse event noted was acute pancreatitis in one patient on the ethanol arm [25]. Similar results were noted in a follow-up study, in which complete resolution was seen in 67% of patients in the ethanol-free arm and 61% of patients in the ethanol arm. Yet again, procedure-related adverse events of pancreatitis and abdominal pain were seen only in the ethanol arm [28]. Therefore, the authors concluded that ethanol was not needed to achieve complete resolution and seemed to be a driver of adverse events following the procedure. 

Two additional studies discussed cytologic and morphologic changes in PCLs after chemoablation with ethanol lavage and/or paclitaxel injection. Kim et al. established multiple EUS changes induced by ablation, including increased diameter, decreased septations, increased epithelial cellularity, and decreased cellular atypia; however, none of these were predictors of cyst resolution [29]. Similarly, An et al. characterized cysts (*n* = 12) that were surgically resected after ablation and noted that many cysts developed eggshell-like calcification on the cyst wall, histiocytic aggregation, ovarian-type stroma, stromal hyalinization, and fat necrosis [30]. 

Paclitaxel is hydrophobic and has a low depot effect due to its high molecular weight and marked protein binding. To overcome these issues, a large surface area microparticle, paclitaxel (LSAM-PTX), has been developed. LSAM-PTX permits suspension formation for targeting drug delivery, allowing delivery of a large dose, and is entrapped at the site of the neoplasm. The drug achieves constant tissue saturation [47,48], releasing paclitaxel into the intended tissue at constant saturation levels. 

**Figure 1:**
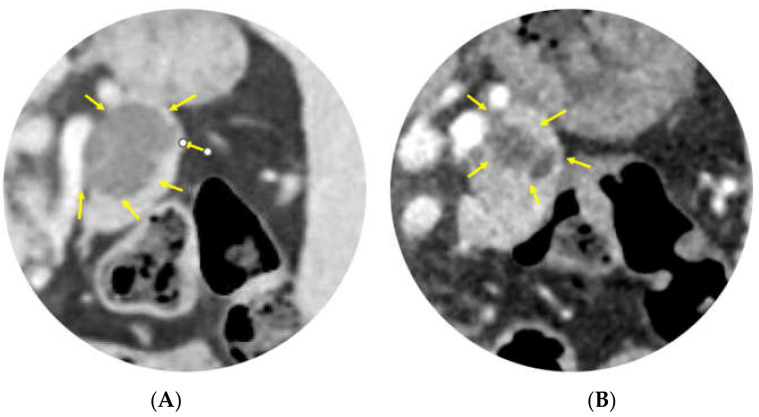
EUS-guided chemoablation with large surface area microparticle paclitaxel (LSAM-PTX) of a branch-duct IPMN. CT-Scan of the abdomen showing a decrease in the lesion size comparing (**A**) pre-ablation to (**B**) post-ablation after 2 injections of LSAM-PTX by EUS-FNI.

This novel formulation of paclitaxel was recently trialed in a multicenter study showing a high safety profile and drug retention in mucinous PCLs [48,49]. The procedure matched that of the previously described studies, such that after aspiration of the cyst, an at least equal volume of LSAM-PTX was injected to create an intralesional depot of paclitaxel. A second phase of the trial was then conducted with the highest dose determined to have good safety and tolerability. A total of 19 patients received LSAM-PTX (the first 9 in the initial dose-escalation phase; 8 received 2 injections 12 weeks apart. There were 17 IPMN and 2 MCN lesions. There were no dose-limiting toxicities or treatment-related serious adverse events, and there was a negligible systemic concentration of paclitaxel. Moreover, there was evidence of intracystic drug retention at 3 months (*n* = 4 patients). There was a response evidenced in 71% of patients with a decrease in cyst size (Figure 1), and 50% of these reductions were at least 30% of the original PCL volume. This early-phase study revealed that EUS-guided chemoablation with LSAM-PTX is safe and allows paclitaxel to continue exerting its effect on the cyst epithelium for months due to its prolonged retention without systemic side effects.

## 6. Durability of Chemoablation

As the various studies described above established the safety and efficacy of EUS-guided chemoablation for the management of PCLs, attention has turned to the durability of this intervention. Choi et al. published a study in 2017 assessing the longer-term outcomes of EUS-EP in a larger study population of 164 patients, including 71 with MCNs, 16 with SCAs, 11 with IPMNs, 3 with pseudocysts, and 3 with indeterminate PCLs. After undergoing the same intervention procedure as used in the previous studies, 114 patients had complete cyst resolution, with partial resolution in 31, and persistent cysts in 13. After the extended follow-up period of an average of 72 months, only 2 of the patients with complete cyst resolution had evidence of recurrence. Two morphologic features of the original PCLs—absence of septa and smaller (≤35 mm) cyst size—predicted resolution [26].

Lester et al. also sought to establish the durability of chemoablation for the management of PCLs by conducting an analysis of patients who participated in the CHARM trial and had follow-up imaging at least 12 months after the original study’s post-treatment response assessment. A radiologist reviewed each patient’s series of imaging, including baseline, 12-month follow-up, and long-term follow-up at least an additional 12 months later. Cysts were measured in multiple dimensions to calculate volume, and the response to EUS-guided chemoablation with paclitaxel and gemcitabine was determined by a reduction in volume. In total, 36 of the original 39 were included in the analysis. The mean long-term follow-up was 36.5 months from the original intervention, with a range of 20–78 months. Of the 23 patients determined to have complete resolution of their PCL in the CHARM trial, 20 demonstrated sustained resolution, whereas the other three had cyst volumes that fell just above the cutoff of 94% reduction in the original volume required to be classified as complete resolution. Additionally, 4 patients who initially had a partial response and 1 who had no response were found to have complete resolution at this later follow-up, while 2 initial non-responders achieved a partial response. Only one patient had a regression from partial response to no response [50]. With this data, the authors concluded that EUS-guided chemoablation is effective in preventing PCL progression and, therefore, has the potential to spare patients from invasive surgical resection. This reinforces the utility of this technique as an option for patients who are unable or unwilling to undergo pancreatectomy.

## 7. Discussion

EUS-guided chemoablation has been shown to be a safe and reliable method of treating premalignant pancreatic cystic lesions. This endoscopic approach is not only less invasive than alternative surgical approaches but is also more cost-effective and associated with significantly fewer adverse effects. The international guidelines that outline the diagnosis of PCLs can be referred to by practicing clinicians [51,52,53,54]. Recognizing the therapeutic impact of EUS-guided interventions, an international position paper was published to address the clinical questions surrounding EUS-guided ablation of PCLs [55]. With the understanding that EUS-guided chemoablation is safe, effective, and well tolerated, recent research efforts are now suggesting how this method can be further improved. The CHARM study successfully challenged the assumption that alcohol lavage was a necessary part of EUS-FNI and demonstrated that it may likely be the inciting factor for common adverse events, including pancreatitis. Various other studies have shown that chemoablation with paclitaxel with or without gemcitabine is an effective injectable agent for achieving the resolution of PCLs with EUS-FNI. However, a large multicenter prospective study with long-term follow-up with standardized procedural techniques is awaited. Current efforts aim to identify optimal concentrations and use molecular engineering to enhance effectiveness. The newest of these innovations is LSAM-PTX, which, in an early phase trial, demonstrated prolonged retention of paclitaxel within PCLs without causing systemic side effects. Another future direction is the application of novel cyst diagnostics, including cyst fluid NGS and EUS-nCLE, and when indicated through needle micro-biopsy. This allows for the accurate diagnosis of the PCL subtype and the risk-stratification of mucinous cysts. These novel diagnostic measures can then be utilized post-ablation to evaluate the response to treatment.

## Figures and Tables

**Table 1:** EUS-guided chemoablation of pancreatic cystic lesions: Published studies with enrollment data, agent used, response rate, adverse events, and study conclusion.

Study	Total Treated	Agent Used	Response Rate	Adverse Effects	Conclusion
Oh et al. 2008 [23]	14 enrolled, 1 cyst unable to be aspirated due to viscosity	Ethanol lavage with paclitaxel injection	Complete resolution in 11 (85%), partial resolution in 2 (15%)	Acute pancreatitis in 1 (8%), hyperamylasemia in 6 (46%), vague abdominal pain in 1 (8%)	Established safety and efficacy of the technique. Cysts with complete resolution had a median volume of 2.75 mL, while the median volume of cysts with partial or no resolution was 5.31 mL
Oh et al. 2009 [24]	10	Ethanol lavage with paclitaxel injection	Complete resolution in 6 (60%), partial resolution in 2 (20%)	Mild pancreatitis in 1 (10%)	Established efficacy of the technique in oligolocular cysts. Cysts with complete resolution had a median volume of 0.54 mL, while cysts with partial or no resolution had 5.07 mL
Oh et al. 2011 [14]	52 enrolled, 47 completed full follow-up period	Ethanol lavage with paclitaxel injection	Complete resolution in 29 (62%), partial resolution in 6 (13%)	Fever without bacteremia in 1 (2%), vague abdominal pain in 1 (2%), pancreatitis in 1 (2%), asymptomatic pericystic spillage in 1 (2%), splenic vein obliteration in 1 (2%)	Established cyst diameter <35 mm (*p* = 0.01) and volume <22 mL (*p* = 0.03) as predictors of cyst resolution. The mean cyst volume was 14.09 mL prior to treatment and 3.31 mL after
DeWitt et al. 2014 [27]	22	Ethanol lavage with paclitaxel injection	Complete resolution in 10 (45%), partial resolution in 5 (23%)	Abdominal pain in 2 (9%), acute pancreatitis in 3 (14%), chemical peritonitis with ileus in 1 (5%), gastric wall cyst in 1 (5%)	Demonstrated efficacy in imaging-based cyst resolution and established elimination of K-ras mutations. Cyst fluid analysis after ablation showed elimination of K-ras mutations in 8 (42%)
Moyer et al. 2016 [25]	Total: 10 Ethanol arm: 4 Ethanol-free arm: 6	Ethanol lavage with paclitaxel vs. normal saline lavage with paclitaxel and gemcitabine injection	Ethanol arm: complete resolution in 3 (75%) Ethanol-free arm: complete resolution in 4 (67%)	Ethanol arm: pancreatitis in 1 (25%) Ethanol-free arm: none	Established similar efficacy with and without ethanol and greater rates of adverse events with ethanol
Moyer et al. 2017 [28]	Total: 39 Ethanol arm: 18 Ethanol-free arm: 21	Ethanol lavage with paclitaxel vs. normal saline lavage with paclitaxel and gemcitabine injection	Ethanol arm: complete resolution in 11 (61%) Ethanol-free arm: complete resolution in 14 (67%)	Ethanol arm: acute pancreatitis in 1 (6%), abdominal pain in 4 (22%) Ethanol-free arm	Affirmed ethanol-free chemoablation as non-inferior and better tolerated than chemoablation with ethanol. Average volume reduction was 78% in the ethanol arm and 84% in the ethanol-free arm
Kim et al. 2017 [29]	Total: 36 Ethanol-only arm: 8 Ethanol and paclitaxel: 28	Ethanol lavage vs. ethanol lavage with paclitaxel injection	Complete resolution in 19 (56%), partial resolution in 7 (21%)	Abdominal pain in 4 (7%), pancreatitis in 4 (7%), intracystic hemorrhage in 1 (2%)	Demonstrated cytologic and morphologic changes induced by ablation, none of which predicted resolution
Choi et al. 2017 [26]	164	Ethanol lavage with paclitaxel injection	Complete resolution in 114 (72%), partial resolution in 31 (20%)	Fever without bacteremia in 1 (<1%), pericystic spillage in 1 (<1%), intracystic hemorrhage in 1 (<1%), portal vein thrombosis in 1 (<1%), splenic vein obliteration in 1 (<1%), main pancreatic duct stricture in 1 (1<%) pseudocyst in 2 (1%), abscess formation in 2 (1%), acute pancreatitis in 6 (4%)	Established durability of the technique with only 2 of those initially showing complete resolution having evidence of recurrence in long-term follow-up (72 months)
An et al. 2022 [30]	12	Ethanol lavage and/or paclitaxel	Partial resolution in 4 (25%)	Not reported	Characterized histopathologic features of surgically-resected pancreatic cysts after ablation

## Data Availability

No new data were created or analyzed in this study. Data sharing is not applicable to this article.
